# Gender Inequalities in Diagnostic Inertia around the Three Most Prevalent Cardiovascular Risk Studies: Protocol for a Population-Based Cohort Study

**DOI:** 10.3390/ijerph18084054

**Published:** 2021-04-12

**Authors:** Concepción Carratala-Munuera, Adriana Lopez-Pineda, Domingo Orozco-Beltran, Jose A. Quesada, Jose L. Alfonso-Sanchez, Vicente Pallarés-Carratalá, Cristina Soriano-Maldonado, Jorge Navarro-Perez, Vicente F. Gil-Guillen, Jose M. Martin-Moreno

**Affiliations:** 1 Clinical Medicine Department, Miguel Hernandez University, 03550 San Juan de Alicante, Spain; maria.carratala@umh.es (C.C.-M.); dorozcobeltran@gmail.com (D.O.-B.); jquesada@umh.es (J.A.Q.); cristina_sori_maldo@hotmail.com (C.S.-M.); vte.gil@gmail.com (V.F.G.-G.); 2 Department of Preventive Medicine and Public Health, School of Medicine, University of Valencia, 46010 Valencia, Spain; Jose.L.Alfonso@uv.es (J.L.A.-S.); jose.martin-moreno@uv.es (J.M.M.-M.); 3 Preventive Medicine Service, General University Hospital Consortium, 46014 Valencia, Spain; 4 Health Surveillance Unit, Castellon Mutual Insurance Union,12004 Castellon, Spain; pallares.vic@gmail.com; 5 Department of Medicine, Jaume I University, 12071 Castellon, Spain; 6 Biomedical Research Institute INCLIVA, Hospital Clinico Universitario de Valencia, University of Valencia, 46010 Valencia, Spain; navarro_jorge@gva.es; 7 Ciber of Epidemiology and Public Health (CIBERESP), Instituto de Salud Carlos III, 28029 Madrid, Spain

**Keywords:** cardiovascular diseases, public health, risk factors, sex factors, disease management

## Abstract

Evidence shows that objectives for detecting and controlling cardiovascular risk factors are not being effectively met, and moreover, outcomes differ between men and women. This study will assess the gender-related differences in diagnostic inertia around the three most prevalent cardiovascular risk factors: dyslipidemia, arterial hypertension, and diabetes mellitus, and to evaluate the consequences on cardiovascular disease incidence. This is an epidemiological and cohort study. Eligible patients will be adults who presented to public primary health care centers in a Spanish region from 2008 to 2011, with hypertension, dyslipidemia, or/and diabetes and without cardiovascular disease. Participants’ electronic health records will be used to collect the study variables in a window of six months from inclusion. Diagnostic inertia of hypertension, dyslipidemia, and/or diabetes is defined as the registry of abnormal diagnostic parameters—but no diagnosis—on the person’s health record. The cohort will be followed from the date of inclusion until the end of 2019. Outcomes will be cardiovascular events, defined as hospital admission due to ischemic cardiopathy, stroke, and death from any cause. The results of this study could inform actions to rectify the structure, organization and training of health care teams in order to correct the inequality.

## 1. Introduction

Cardiovascular disease (CVD) is still the main cause of death worldwide [1]. Previous investigations have identified differences in the management of these diseases according to gender; correcting these inequalities could help to improve prevention, diagnosis, and treatment in both men and women [2,3]. Although CVD affects a larger proportion of women than men [4,5,6], it is still considered a man’s disease [2,7]. Different studies have shown that women are less likely to receive preventive treatment or behavioral recommendations according to guidelines than men with a similar risk [8,9]. Moreover, treatment intensification and the achievement of therapeutic targets are less frequent in women [10,11]. This tendency may be due to differences related to both clinical presentation and the perception that women are “protected” against CVD until menopause [3]. The gender differences in the diagnosis and management of CVD have mainly been studied in the hospital setting, while data from the primary health care context are more limited.

CVD is amenable to prevention through population-based interventions targeted to modifiable risk factors, like tobacco use, obesity, physical inactivity, or excessive alcohol intake. In people at high cardiovascular risk, additional measures related to early detection and treatment of classical factors like hypertension, diabetes, and dyslipidemia are crucial [2]. The prevalence of these risk factors is generally similar in men and women, but their presentation or effects may be qualitatively or quantitatively different. For example, diabetes increases the risk of coronary disease more in women than in men [12], while metabolic syndrome is the most important risk factor for very early-onset ischemic cardiopathy in women [13]. Similarly, women who smoke are more likely to have coronary ischemia compared to men [14], and while women develop hypertension and dyslipidemia later than men, the control of these factors is worse in women [15,16]. At the same time, women are also affected by different cardiovascular risk factors, such as premature childbirth, pre-eclampsia, and gestational diabetes [8].

The last decade has seen a growing understanding of the need to focus on gender-related differences in CVD prevention, diagnosis, and treatment [8]. The American Heart Association (AHA) has published evidence-based guidelines on the primary prevention of CVD in women, first in 2007 and then as an update in 2011 [17]; these recommendations have promoted a change in the clinical research on this topic [8]. But despite improvements, the care women receive and their health outcomes still differ compared to men. Moreover, there has been little action to address gender inequalities in CVD care, in part because the quality of care is not measured or presented by gender. Greater awareness in the identification and control of individuals’ risk factors according to this variable could improve the prevention of cardiovascular events [3].

Current clinical practice guidelines [18,19,20] contain strategies for the primary prevention of CVD and the diagnosis and management of hypertension, diabetes, dyslipidemia, and other cardiovascular risk factors in both men and women. However, the evidence shows that goals for detecting and controlling these risk factors are not being adequately met.

One of the main reasons for this lack of control is clinical inertia, a concept first defined by Phillips in 2001 as the failure of physicians to initiate or intensify treatment when this is indicated [21]. Subsequently, this concept has been redefined as therapeutic inertia, while clinical inertia has broadened to encompass additional aspects, including the lack of diagnosis or follow-up. Gil-Guillen et al. [22] differentiated diagnostic inertia from therapeutic inertia in the case of patients whose medical records showed sustained levels of elevated blood pressure but had not been diagnosed or treated for hypertension.

Clinical inertia is common in pathologies like dyslipidemia or arterial hypertension. Previous studies have shown that this factor has resulted in the failure to diagnose 65.3% of lipid alterations [23], and it is present in one of every three cases of high blood pressure in the Valencian Community [22]. These results highlight the need for a multifactorial focus to address this problem, including actions on the structure, organization, and training of health care teams [22].

To our knowledge, the potential gender-related differences in diagnostic inertia are not well delineated in the literature, which may be due to the recent definition of the phenomenon. However, there are studies about gender differences in therapeutic inertia; in 2007, Chou et al. [24] observed inadequate levels of low-density lipoprotein (LDL) cholesterol in both men and women, but with lower levels of control in women. This result suggests that treatment may also be less intensive in women, that is, with greater therapeutic inertia in this group. A better understanding of the different health care patterns in men and women could therefore aid in efforts to prevent CVD [25]. Thus, the overarching aim of this study is to assess the gender-related differences in diagnostic inertia around the three most prevalent cardiovascular risk factors: dyslipidemia, arterial hypertension, and diabetes mellitus, and to evaluate its relationship with the risk of a cardiovascular event in adults aged 30 or over who have one of these risk factors.

## 2. Materials and Methods

### 2.1. Study Design and Population

This is an epidemiological ambispective cohort study in the Spanish region of the Valencian Community. Study participants from a previous cohort [25] will be included in the present study. The Estudio Cardiometabólico Valenciano (ESCARVAL) study [25,26,27,28,29,30] was a prospective cohort study and patients who presented to public primary health care centers in the Valencian Community, without clinical CVD but at least one of the following cardiovascular risk factors: dyslipidemia, arterial hypertension, and diabetes mellitus (whether diagnosed or with abnormally high control indicators) were recruited between 1 January, 2008 and 31 December, 2011. In the present study, eligible patients will be men and women aged 30 years or more who were included in the ESCARVAL study [19] and who were followed using the electronic health record (EHR) under the normal conditions of clinical practice.

### 2.2. Study Variables

The window of exposure is defined as the six months starting from study inclusion; the exposure variable is diagnostic inertia. For hypertension, this will be defined according to clinical practice guidelines as high registered levels of systolic (≥140 mmHg) or diastolic (≥90 mmHg) blood pressure on two separate occasions during the window of exposure, but without a diagnosis of arterial hypertension, based on the corresponding code from the International Classification of Diseases, 9th edition (ICD-9: 401–405). Diagnostic inertia of dyslipidemia is defined as no diagnosis (ICD-9: 272) despite blood tests showing abnormally high lipids at any time point in the window of exposure, established by clinical guidelines as levels of total cholesterol of more than 200 mg/dL or levels of high-density lipoprotein (HDL) cholesterol of less than 45 mg/dL. Finally, diagnostic inertia of diabetes is defined as the lack of a diagnosis of diabetes mellitus (ICD-9: 250) in the presence of at least two measurements of elevated fasting glycemia (≥126 mg/dL) or glycated hemoglobin (HbA1c ≥ 6.5%) on two separate occasions during the window of exposure.

Other parameters of interest include physical (age, sex), clinical (body mass index (BMI), abdominal circumference, diastolic and systolic blood pressure), behavioral (tobacco use and alcohol intake), and analytical (HbA1c; glycemia; non-HDL, HDL, and LDL cholesterol; triglycerides, creatinine, glomerular filtration rate, albuminuria, and uric acid) indicators. We will also collect variables related to pharmacological treatment (antihypertensive drugs, statins, and other lipid-lowering drugs, oral antidiabetic drugs, insulin, and antithrombotics) and to damage in the target organ (cardiac insufficiency, atrial fibrillation, left ventricular hypertrophy, chronic kidney disease, proteinuria, and retinopathy).

Participants’ EHRs will be used to collect study variables. The validity of laboratory data will be ensured thanks to the existence of an online laboratory system, in which an analyst in each reference hospital enters the data, obviating the need for a manual registry in the primary health care clinic. The data sources for recording cardiovascular events are the Valencian Community Mortality Registry and the hospital discharge report from the minimum basic data set.

### 2.3. Study Follow-Up

The cohort will be followed from 2008 to 2019 (inclusive), with the outcome variable of all-cause mortality (with the date of death) obtained from an annual cross-check with the mortality registry. Hospital admissions and their dates will be recorded based on the minimum basic data set, using the appropriate ICD codes for ischemic cardiopathy (ICD-9: 410–414), ischemic stroke (ICD-9: 434.91), or intracranial hemorrhage (ICD-9: 430–432). This information of these databases is not current: data from 2019 will only be received in 2021, the year that this study is set to end.

### 2.4. Outcomes

**Figure 1:**
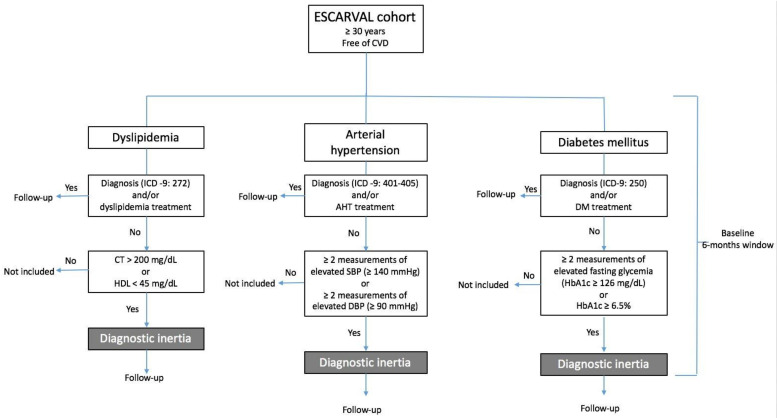
Study procedures.

The primary outcome variable will be the first occurrence of a cardiovascular event (morbimortality) during the study period, defined as a hospital admission due to ischemic cardiopathy, stroke, and death from any cause. Figure 1 shows the study procedures.

### 2.5. Statistical Analysis

Data analysis will be performed using the IBM SPSS Statistics for Windows, v. 26.0. (Armonk, NY: IBM Corp) and R software, v.4.0.2 (R Core Team, 2020). Quantitative variables will be expressed as means and standard deviations, and qualitative variables as absolute and relative frequencies, with their corresponding 95% confidence intervals (CIs). To measure crude associations between qualitative variables, we will construct 2 × 2 tables and apply the chi-squared test. For quantitative variables, the normality of the distribution will be analyzed, and groups will be compared using the student *t*-test, ANOVA test, or non-parametric tests, as appropriate. To estimate the magnitude of the cardiovascular risk according to both diagnostic inertia and to other variables of interest, Cox proportional regression models will be fit to estimate the hazard ratio and 95% CI for cardiovascular events. The parameters included in the multivariable model will be selected using the stepwise method, based on the Akaike information criterium. We will test the hypothesis of proportional hazards, adjusting time-dependent components if the hypothesis is not met. Possible interactions between the factors of interest will be considered. For the statistical analysis, single blinding will be used to mask the gender of the analyzed participants to prevent any potential gender bias in the analysis.

## 3. Discussion

Our study hypothesis is that there are gender-related differences in diagnostic inertia. The analysis of gender inequalities derived from diagnostic inertia in the three most prevalent cardiovascular risk factors and its relationship with the risk of a cardiovascular event in the general population could inform strategies for CVD prevention in groups with risk factors that are not being adequately diagnosed. Clinical inertia is frequent in chronic cardiovascular pathologies, highlighting the need for a multifactorial focus to tackle the problem, including actions targeted to the structure, organization, and training of health care teams. This study is a first step for understanding the influence of diagnostic inertia and gender differences as well as the control of cardiovascular risk factors on the incidence of cardiovascular events.

In the Valencian Community, a European region with a population of 5 million, every person has a single EHR collecting all data from primary, secondary, and tertiary care. The system, called ABUCASIS, is a valuable resource, enabling the performance of this kind of study, similarly to other EHR-based information systems internationally, like the THIN database in the UK’s National Health System [31]. The initial results of the ESCARVAL study are already available [25,26,27,28,29,30], which provide interesting results after five years’ follow-up but which need to be confirmed and contrasted with more long-term outcomes.

The results of this observational study should be interpreted in light of the advantages and limitations of the registry-based data it uses [32]. EHRs are a valuable source of data, reflecting real-world clinical practice in regular patients in the primary care setting, in contrast with the data from clinical trials, which do not correspond with routine practice and whose selected participants are not always representative of the population. Moreover, EHR databases allow access to longitudinal data in large populations at a low cost, which is very useful for epidemiological research. Another potential advantage is the large number of participants and events that can be studied that provide sufficient statistical power and a valuable framework for evaluating cardiovascular risk in real-world situations. Previous studies have been shown to exaggerate or ignore gender-related inequalities in outcomes. The present study has the advantage of being specifically designed to detect these possible differences and their influence on the incidence of cardiovascular events. To avoid any potential gender bias, the gender of study participants will be masked.

## 4. Conclusions

The information that this study will provide could be essential for improving medical and nursing care in the primary health care setting, and it could identify gender-related inequalities in the diagnosis and management of risk factors associated with CVD, enabling the prevention of future disease. As a population-based study, it could contribute to general improvements in the efficiency of the health care system. Moreover, in the medium term, it could help to generate new working hypotheses in this area, laying the foundation for future research studies of gender bias in the management of CVD in national and international contexts.

## Data Availability

Data sharing is not applicable to this study protocol.
